# The effect and relative importance of sleep disorders for all-cause mortality in middle-aged and older asthmatics

**DOI:** 10.1186/s12877-022-03587-2

**Published:** 2022-11-14

**Authors:** Zhigang Hu, Yufeng Tian, Xinyu Song, Fanjun Zeng, Ke Hu, Ailan Yang

**Affiliations:** 1 Department of Respiratory and Critical Care Medicine, The first College of Clinical Medicine Science, China Three Gorges University, Yichang, 443003 China; 2 Department of Respiratory and Critical Care Medicine, Zhijiang People’s Hospital, Yichang, 443003 China; 3 Department of Respiratory and Critical Care Medicine, Yichang Central People’s Hospital, Yichang, China; 4 Department of Respiratory and Critical Care Medicine, the first College of Clinical Medicine Science, Three Gorges University, 183 Yiling Road, Yichang, 443003 China; 5 Department of Respiratory and Critical Care Medicine, Renmin Hospital of Wuhan University, Wuhan, China

**Keywords:** Sleep disorder, Sleep duration, Excessive daytime sleepiness, all-cause mortality; asthma.

## Abstract

**Background:**

Previous studies observed that **s**leep disorders potentially increased the risk of asthma and asthmatic exacerbation. We aimed to examine whether excessive daytime sleepiness (EDS), probable insomnia, objective short sleep duration (OSSD), and obstructive sleep apnea (OSA) affect all-cause mortality (ACM) in individuals with or without asthma.

**Methods:**

We extracted relevant data from the Sleep Heart Health Study established in 1995–1998 with an 11.4-year follow-up. Multivariate Cox regression analysis with a proportional hazards model was used to estimate the associations between ACM and four sleep disorders among asthmatic patients and individuals without asthma. Dose-response analysis and machine learning (random survival forest and CoxBoost) further evaluated the impact of sleep disorders on ACM in asthmatic patients.

**Results:**

A total of 4538 individuals with 990 deaths were included in our study, including 357 asthmatic patients with 64 deaths. Three multivariate Cox regression analyses suggested that OSSD (adjusted HR = 2.67, 95% CI: 1.23–5.77) but not probable insomnia, EDS or OSA significantly increased the risk of ACM in asthmatic patients. Three dose-response analyses also indicated that the extension of objective sleep duration was associated with a reduction in ACM in asthmatic patients compared to very OSSD patients. Severe EDS potentially augmented the risk of ACM compared with asthmatics without EDS (adjusted HR = 3.08, 95% CI: 1.11–8.56). Machine learning demonstrated that OSSD of four sleep disorders had the largest relative importance for ACM in asthmatics, followed by EDS, OSA and probable insomnia.

**Conclusions:**

This study observed that OSSD and severe EDS were positively associated with an increase in ACM in asthmatic patients. Periodic screening and effective intervention of sleep disorders are necessary for the management of asthma.

## Introduction

Many epidemiological studies have suggested that sleep disorders are associated with an increase in the morbidity and mortality of some metabolic disorders and cardiovascular diseases [1–4]. Sleep disorders mainly include excessive daytime sleepiness (EDS), insomnia disorder, obstructive sleep apna (OSA), short sleep duration, etc. The healthy sleep duration of adults is preferred to be between 7 h and 9 h per night throughout the world [3, 4]. However, this indication does not correspond to real-world life. Approximately 35.0% of individuals in U.S. A, 11.3% in Canada and 9.8% in the U. K had sleep insufficiency (sleep duration ≤6 h per night) [5, 6]. A previous study suggested that asthma in stable condition leads to a reduction of approximately 50 minutes of sleep duration [7]. A recent study demonstrated that asthmatics were associated with 43% of high risk for OSA, 25% of EDS and 46.5% of clinical insomnia [8], which seemed to be more common than the general population [9, 10].

Emerging evidence demonstrates that sleep disorders play a crucial role in the development and exacerbation of asthma. Previous studies demonstrated that short sleep duration is associated with a significantly higher prevalence of asthma [11] and asthmatic attack [12] than healthy sleep duration. Other studies have shown that a reduction in sleep duration is associated with a higher asthma treatment step [13] and worse lung function compared with healthy sleep duration [14]. However, there is no published study about the association between asthmatic mortality and objective sleep duration.

Cumulative evidence about the interaction between OSA and asthma suggests that asthma may increase the prevalence of OSA-related symptoms [15, 16] and polysomnographically diagnosed OSA [16]. OSA has an important contribution to the severity of asthma [17] and hospital length of stay secondary to asthmatic exacerbation [18]. However, OSA seemingly has no significant impact on in-hospital mortality [18] or all-cause mortality (ACM) among asthmatic patients [19, 20]. No published study reports the association between the severity of OSA and ACM in asthmatic patients. The study indicated that EDS and insomnia were potential risk factors for the severity of asthma [21]. Regrettably, no study has estimated the impact of EDS and insomnia on ACM among asthmatic patients.

Our previous studies explored the associations between short sleep duration and the prevalence [11], episode [12], phenotype [14], fractional exhaled nitric oxide (FeNO) [14], and lung function [14] of asthma. The focus of this study was to estimate the effect of sleep disorders on ACM in asthmatic patients. The first step examined whether four sleep disorders cause different ACM results in individuals with and without asthma. The second step further assessed the associations between ACM of asthmatics and the severity of EDS, OSA, and OSSD through dose-dependent analysis In addition, machine learning was used to evaluate the relative importance of four sleep disorders for ACM in asthmatic patients.

## Methods

### Study design and population

The Sleep Heart Health Study is a prospective multicenter cohort study of the population aged ≥40 years old implemented by the National Heart Lung & Blood Institute. This study planed to explore the association between cardiovascular consequences and sleep-disordered breathing. The recruitment of individuals came from nine existing epidemiological studies in which data on cardiovascular risk factors had been collected previously [22]. In the baseline assessments, 5804 individuals between November 1995 and January 1998 were enrolled with an initial polysomnogram. Trained and certified technicians performed polysomnograms in the homes of the individuals. 3295 individuals received the second polysomnogram between January 2001–June 2003. Cardiovascular outcomes and ACM surveillance with about 11.4 years follow-up and two exam cycles were continued until 2010. More detailed information about this study has been published elsewhere [22] and and the following link: https://sleepdata.org/datasets/shhs.

### Interviews and measurements

Physician-diagnosed asthma was diagnosed according to the following questions: “Doctor of Medicine said participant had asthma?”. For patients with asthma, we also collected the data about inhaled steroids for asthma and asthmatic attack based on two following questions: “Participant taking inhaled steroids for asthma within two weeks of SHHS?” (inhaled steroids for asthma) and “Participant had an attack of asthma in the last 12 months?” (asthmatic attack). The following variables were used to adjust the potential association between sleep disorders and ACM: age, sex, race, body mass index (BMI), smoking status, coffee consumption, comorbidities (diabetes, hypertension, angina, myocardial infarction, chronic obstructive pulmonary disease (COPD) and high cholesterol), and drug use (benzodiazepines, tricyclic antidepressants and nontricylic antidepressants).

By using questionnaires, we obtained data on Epworth Sleepiness Scale (ESS) scores and the diagnosis of probable insomnia. ESS scores > 10 were regarded as the diagnosis of EDS. According to ESS scores, EDS was divided into three groups: mild (11–12 scores), moderate (13–15 scores) and severe (≥ 16 scores) [9]. The diagnosis of probable insomnia simultaneously included the symptoms and daytime consequences of insomnia [23]. People with one or more of “trouble falling asleep”, “waking up and having difficulty resuming sleep”, and “waking up too early and unable to resume sleep” (frequency = 16–30 nights/months) were regarded as having insomnia symptoms [24]. Individuals with “frequency of feeling unrested ≥ 5-15 times/months” or “frequency of excessive daytime sleepiness ≥ 5-15 times/months” were considered adverse daytime consequences with reference to a previous study [24]. Polysomnogram in the home provided objective sleep data. Because individuals with objective sleep duration ≥480 minutes were very few, objective sleep duration was categorized into four groups to perform dose-response analysis with reference to a previous study: ≥ 420 minutes (healthy), 360–419 minutes (relatively healthy), 300–359 minutes (short), and < 300 minutes (very short) [25]. Objective sleep duration refers to total sleep time with short sleep duration < 6 hours. Wake after sleep onset and sleep efficiency were considered potentially confounding factors [26]. Sleep stage was divided into four groups (N1, N2, N3, and rapid eye movement (REM) sleep stages), and the percentages of time spent were recorded. A decreased percentage of REM sleep was found to increase mortality [26]; thus, it was included as an adjusted variable. Meanwhile, the authors obtained the following data about hyoxemia: the percentage of time spent in sleep below 90% oxygen saturation (T90%). The apnea hypopnea index (AHI) was defined as the number of obstructive apnea plus hypopneas associated with ≥3% oxygen desaturation per hour of sleep. According to the AHI, OSA was classified as mild (AHI = 5–14.9 events/h), moderate (AHI = 15–29.9 events/h) or severe (AHI ≥ 30 events/h). Lung function included forced expiratory volume in 1second (FEV1), forced vital capacity (FVC), and FEV1/FVC ratio.

### Statistical analysis

Categorical variables are presented as counts and percentages (%) with a chi-square test. Means and standard deviations were used to express continuous variables with the Mann-Whitney U test for skewed continuous variables and Student’s t test or one-way ANOVA for normally distributed continuous variables. The normality of the distribution of the data was tested by the chi-square goodness-of-fit test.

Multivariate Cox regression analysis with a proportional hazards model was used to evaluate the associations between ACM and sleep disorders in the three models. We checked the proportional hazards assumption by using statistical tests and graphical diagnostics on the basis of the scaled Schoenfeld residuals. The variables included in the three models are listed in the table. Model 1 included patient demographic and behavioral factors, model 2 included comorbidities and drug use, and model 3 included comorbidities, drug use, FEV1, FEV1/FVE, wake after sleep onset, sleep efficiency, TIMEREMP, and T90. In the second step of the study, inhaled steroids for asthma and asthma attack were adjusted. EDS, OSA and objective sleep duration were grouped and used to perform dose-dependent analysis in three models. Machine learning has the ability to better deal with high-dimensional data and determine complex associations between variables and clinical outcomes compared with traditional statistical analysis with the limitation of special assumptions [27]. The study population was divided into a training and hold-out test set at an 80/20 ratio. At present, random survival forest and CoxBoost machine learning are available to perform survival analysis. A random survival forest may measure the relative importance of variables for clinical outcomes through the variable importance (VIMP) method and minimal depth method [28]. CoxBoost can provide standardized coefficient estimates for variables in survival analysis. The closer the standardized coefficient becomes zero, the less the effect of the variable on clinical outcomes.

Stata 14, R, and Empower(R) software (www. empowerstats.com; X&Y solutions, Inc., Boston MA) were used to complete all statistical analyses. The hazard ratio (HR) with 95% CI was used to estimate the differences, and a two-tailed *P* < 0.05 was considered statistically significant.

## Results

### The demographic and clinical characteristics of the study population

A total of 4538 individuals who were predominantly female (51.8%) and white (87.7%) were included in this study. There were 30% of individuals with obesity (BMI ≥ 30 kg/m2) and 56.4% with high blood cholesterol (≥200 mg/dl). The prevalence of hypertension and airway obstruction (FEV1/FVC < 0.7) was 42.2 and 18.7%, respectively. The mean ESS scores, objective sleep duration, and AHI were 7.8, 361 minutes, and 14.7 events/h, respectively. Approximately 5 and 24.9% of individuals were diagnosed with probable insomnia and EDS, respectively. The percentages of OSSD and OSA in the study population were 44.3 and 69.7%, respectively. In our study, the study population was stratified by physician-diagnosed asthma. The asthma group included 357 individuals with 64 deaths, whereas the no-asthma group comprised 4181 individuals with 926 deaths at a follow-up time of approximately 11.4 years. The mean values of age and FEV1 in the asthma group were lower than those in the no-asthma group. The prevalence of probable insomnia and EDS in the asthma group was significantly higher than that in the no-asthma group. The more detailed demographic and clinical characteristics of the study population are shown in Table 1.

**Table 1:** The characteristics of study population without and with asthma

	Without asthma	With asthma	P
N	4181	357	
Sex			< 0.01
Male	2042 (48.8%)	147 (41.2%)	
Female	2139 (51.2%)	210 (58.8%)	
Age			< 0.01
< 50 years	432 (10.3%)	64 (17.9%)	
50 to 59 years	1120 (26.8%)	102 (28.6%)	
60 to 69 years	1263 (30.2%)	94 (26.3%)	
70 to 79 years	1075 (25.7%)	80 (22.4%)	
≥ 80 years	291 (7.0%)	17 (4.8%)	
Race			< 0.01
White	3685 (88.1%)	297 (83.2%)	
Black	253 (6.1%)	39 (10.9%)	
Other	243 (5.8%)	21 (5.9%)	
Smoking status			0.63
Never	1992 (47.6%)	178 (49.9%)	
Current	376 (9.0%)	28 (7.8%)	
Former	1813 (43.4%)	151 (42.3%)	
Coffer consumption (cups/day)			< 0.01
0	1643 (39.3%)	164 (45.9%)	
1	648 (15.5%)	61 (17.1%)	
2	734 (17.6%)	64 (17.9%)	
≥ 3	1156 (27.6%)	68 (19.0%)	
Body mass index (BMI, kg/m2)			0.62
< 25	1168 (27.9%)	94 (26.3%)	
25 to 29.9	1765 (42.2%)	148 (41.5%)	
≥ 30	1248 (29.8%)	115 (32.2%)	
Diabetes			0.67
No	3890 (93.0%)	330 (92.4%)	
Yes	291 (7.0%)	27 (7.6%)	
Hypertension			0.94
No	2416 (57.8%)	207 (58.0%)	
Yes	1765 (42.2%)	150 (42.0%)	
Angina			0.90
No	3884 (92.9%)	331 (92.7%)	
Yes	297 (7.1%)	26 (7.3%)	
Myocardial infarction			0.18
No	3905 (93.4%)	340 (95.2%)	
Yes	276 (6.6%)	17 (4.8%)	
COPD			< 0.01
No	4158 (99.4%)	335 (93.8%)	
Yes	23 (0.6%)	22 (6.2%)	
High blood cholesterol(≥200 mg/dl)			0.70
No	1818 (43.5%)	159 (44.5%)	
Yes	2363 (56.5%)	198 (55.5%)	
Benzodiazepines use			0.50
No	3959 (94.7%)	341 (95.5%)	
Yes	222 (5.3%)	16 (4.5%)	
Tricylic anti-depressants use			0.04
No	4073 (97.4%)	341 (95.5%)	
Yes	108 (2.6%)	16 (4.5%)	
Non-tricyclic antidepressants use			< 0.01
No	4007 (95.8%)	323 (90.5%)	
Yes	174 (4.2%)	34 (9.5%)	
FEV1	2.7 ± 0.8	2.4 ± 0.8	< 0.01
FEV1/FVC1			< 0.01
< 0.7	708 (16.9%)	141 (39.5%)	
≥ 0.7	3473 (83.1%)	216 (60.5%)	
Probable insomnia			0.02
No	3976 (95.1%)	329 (92.2%)	
Yes	205 (4.9%)	28 (7.8%)	
ESS score	7.7 ± 4.4	8.5 ± 4.3	< 0.01
Excessive daytime sleepiness (ESS > 10)			0.12
Normal (ESS ≤ 10)	3156 (75.5%)	250 (70.0%)	
Mild (ESS = 11–12)	428 (10.2%)	47 (13.2%)	
Moderate (ESS = 13–15)	362 (8.7%)	34 (9.5%)	
Severe (ESS ≥ 16)	235 (5.6%)	26 (7.3%)	
Wake after sleep onset			0.82
< 30 minutes	1043 (24.9%)	91 (25.5%)	
≥ 30 minutes	3138 (75.1%)	266 (74.5%)	
Sleep efficiency			0.16
< 0.8	1289 (30.8%)	123 (34.5%)	
≥ 0.8	2892 (69.2%)	234 (65.5%)	
TIMESN1P	5.4 ± 3.8	5.1 ± 3.6	0.13
TIMESN2P	56.5 ± 11.5	55.9 ± 12.2	0.34
TIMESN3P	18.1 ± 11.7	19.2 ± 12.2	0.09
TIMEREMP	20.0 ± 6.1	19.8 ± 6.5	0.61
Objective sleep duration (minutes)	361.9 ± 63.2	359.8 ± 65.0	0.56
Objective sleep duration (minutes)			0.55
Very short(< 300)	663 (15.9%)	59 (16.5%)	
Short(300–359)	1181 (28.2%)	106 (29.7%)	
Relatively healthy(360–419)	1600 (38.3%)	123 (34.5%)	
Healthy(≥ 420)	737 (17.6%)	69 (19.3%)	
T90	3.5 ± 10.4	3.0 ± 8.8	0.43
AHI, events/h	14.3 ± 14.8	12.7 ± 13.4	0.04
Obstructive sleep apnea			0.14
Normal (AHI < 5)	1264 (30.2%)	110 (30.8%)	
Mild (AHI = 5–14.9)	1486 (35.5%)	145 (40.6%)	
Moderate (AHI = 15–29.9)	912 (21.8%)	66 (18.5%)	
Severe (AHI ≥ 30)	519 (12.4%)	36 (10.1%)	
Follow-up time (days)	4016.0 ± 1107.5	4128.3 ± 1114.7	0.07
Survival status			0.06
Alive	3255 (77.9%)	293 (82.1%)	
Dead	926 (22.1%)	64 (17.9%)	

Note: AHI, Apnea Hyponea Index; COPD, Chronic obstructive pulmonary disease: ESS, Epworth Sleepiness Scale; TIMESN1P, Percent of sleep time in stage 1 sleep; TIMESN2P, Percent of sleep time in stage 2 sleep; TIMESN3P, Percent of sleep time in stage 3/4 sleep; TIMEREMP, Percent of sleep time in rapid eye movement sleep.

#### The associations between all-cause mortality and sleep disorders

In the asthma group, all three multivariate regression analyses suggested that healthy sleep duration was associated with a lower risk of ACM than OSSD (adjusted HR = 2.67, 95% CI: 1.23–5.77, *P* = 0.01 in model 3). Probable insomnia, EDS, and OSA seemingly had no significant effect on the ACM of asthmatic patients in the three models (see Table 2).

**Table 2:** The associations between sleep disorders and all-cause mortality among study population without and with asthma

	Model 1	*P*	Model 2	*P*	Model 3	*P*
Study population with asthma						
EDS	1.40(0.78, 2.52)	0.26	1.24(0.67, 2.27)	0.49	1.26(0.64, 2.48)	0.50
Probable insomnia	0.64(0.23, 1.75)	0.38	0.54(0.18, 1.6)	0.27	0.46(0.14,1.52)	0.20
OSA	1.20(0.62, 2.31)	0.58	1.15(0.58, 2.31)	0.68	1.10(0.48, 2.53)	0.82
OSSD	2.64(1.44, 4.8)	< 0.01	3.10(1.63, 5.92)	< 0.01	2.67(1.23, 5.77)	0.01
Study population without asthma						
EDS	0.87(0.74, 1.02)	0.08	0.83(0.71,0.97)	0.02	0.85(0.73, 1.01)	0.05
Probable insomnia	1.47(1.10, 1.97)	0.01	1.36(1.01, 1.84)	0.04	1.37(1.02, 1.86)	0.04
OSA	1.20(1.02, 1.42)	0.03	1.15(0.98, 1.36)	0.09	0.98(0.81,1.18)	0.85
OSSD	1.05(0.92, 1.20)	0.43	1.05(0.92, 1.20)	0.5	0.91(0.79, 1.06)	0.24

Note: EDS, Excessive daytime sleepiness (ESS > 10 score); OSA, Obstructive sleep apnea (AHI ≥ 5 events/h); OSSD, Objective short sleep duration (Objective sleep duration< 360 minutes);

Model 1 included sex, age, race, smoking status, coffee consumption, BMI, excessive daytime sleepiness, probable insomnia, obstructive sleep apnea and objective sleep duration.

Model 2 = Model1+ diabetes, hypertension,angina, myocardial infarction, COPD, high cholesterol, and drugs use (benzodiazepines, tricylic anti-depressants and non-tricylic anti-depressants).

Model 3 = Model 2 + FEV1, FEV1/FVE, wake after sleep onset, sleep efficiency, TIMEREMP, T90.

In individuals without asthma, model 1 indicated that the presence of probable insomnia (adjusted HR = 1.47, 95% CI: 1.10, 1.97, *P* = 0.01) and OSA (adjusted HR = 1.20, 95% CI: 1.02, 1.42, *P* = 0.03) potentially increased the risk of ACM compared with the reference group. However, the association between ACM and OSA became insignificant after adjusting for comorbidity and drug use in model 2 (see Table 2). In model 3, sleep disorders, with the exception of probable insomnia, were not significantly associated with ACM when adjusted for all confounding factors (see Table 2).

#### Dose-dependent analysis of the associations between all-cause mortality and sleep disorders

**Fig. 1:**
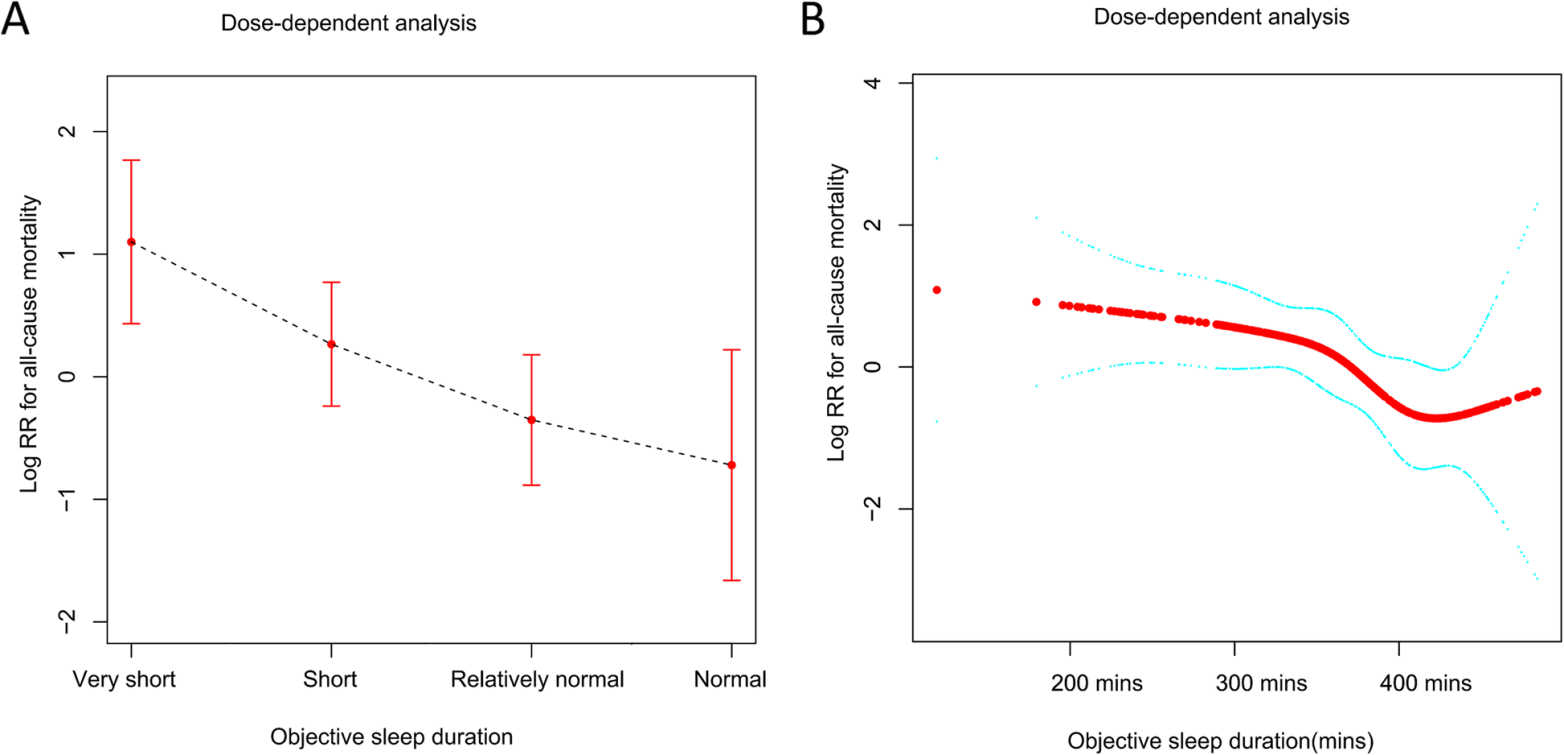
Log hazard ratio of all-cause mortality as a smooth function of exposure to objective sleep duration among asthmatics, as estimated from the Cox proportional hazards model. (A) Objective sleep duration was regarded as a categorical variable; (B) objective sleep duration was seen as a continuous variable

In the asthma group, three multivariate Cox regression analyses demonstrated that the cumulative incidences of ACM in 300–359 minutes, 360–419 minutes, and ≥ 420 minutes of objective sleep duration were significantly lower during follow-up with the adjustment of all included risk factors (see Table 3) compared with very OSSD (< 300 minutes). Figure 1 graphically displays the log hazard risk of ACM as a smooth function of cumulative exposure to objective sleep duration. When objective sleep duration was regarded as a categorical variable, the hazard risk of ACM declined with the increase in objective sleep duration among asthmatic patients (see Fig. 1A). When objective sleep duration was seen as a continuous variable, approximately 420 minutes of sleep was associated with the lowest hazard risk of ACM (see Fig. 1B). More than 420 minutes of objective sleep duration seemingly had a trend of increasing ACM. Model 2 (adjusted HR = 2.78, 95% CI: 1.11, 6.86, *P* = 0.03) and model 3 (adjusted HR = 3.08, 95% CI: 1.11, 8.56, *P* = 0.03) showed that individuals with severe EDS harbored significantly higher ACM than those without EDS. Probable insomnia and the severity of OSA seemingly had no significant impact on ACM in the three models (see Table 3). In the no asthma group, model 3 showed that the other three groups of OSSD, EDS, and OSA were not associated with significantly different ACMs compared with the control group.

**Table 3:** Dose-dependent analyses of the associations between sleep disorders and all-cause mortality among study population with asthma

	Model 1	*P*	Model 2	*P*	Model 3	*P*
Excessive daytime sleepiness						
Normal	Ref		Ref		Ref	
Mild	1.87(0.85, 4.12)	0.12	1.69(0.73, 3.88)	0.22	2.05(0.83, 5.03)	0.12
Moderate	0.44(0.12, 1.63)	0.22	0.47(0.13, 1.75)	0.26	0.57(0.14, 2.27)	0.43
Severe	2.17(0.91, 5.15)	0.08	2.78(1.11, 6.86)	0.03	3.08(1.11, 8.56)	0.03
Probable insomnia						
No vs yes	0.48(0.17, 1.37)	0.17	0.37(0.12, 1.14)	0.08	0.28(0.08, 1.00)	0.05
Obstructive sleep apnea						
Normal	Ref		Ref		Ref	
Mild	1.19(0.61, 2.33)	0.62	0.94(0.45, 1.97)	0.88	0.97(0.41, 2.27)	0.94
Moderate	1.54(0.66, 3.58)	0.31	1.83(0.77, 4.38)	0.17	1.55(0.5, 4.75)	0.45
Severe	0.79(0.26, 2.44)	0.68	0.57(0.17, 1.88)	0.36	0.44(0.1, 1.92)	0.27
Objective sleep duration						
Very short	Ref		Ref		Ref	
Short	0.46(0.24, 0.88)	0.02	0.43(0.21, 0.88)	0.02	0.44(0.21, 0.94)	0.03
Relatively healthy	0.25(0.12, 0.52)	< 0.01	0.22(0.10, 0.50)	< 0.01	0.25(0.09, 0.69)	< 0.01
Healthy	0.23(0.08, 0.65)	< 0.01	0.15(0.05, 0.39)	< 0.01	0.19(0.05, 0.69)	0.01

Note: Model1 included sex, age, race, smoking status, coffee consumption, BMI, excessive daytime sleepiness, probable insomnia, obstructive sleep apnea and objective sleep duration.

Model 2 = Model 1+ diabetes, hypertension, angina, myocardial infarction, COPD, high cholesterol, drugs use (benzodiazepines, tricylic anti-depressants and non-tricylic anti-depressants), inhaled steroids for asthma, asthmatic attack.

Model 3 = Model 2 + FEV1, FEV1/FVC, wake after sleep onset, sleep efficiency, TIMEREMP, T90.

#### Machine learning of the associations between all-cause mortality and sleep disorders in the asthma group

**Fig. 2:**
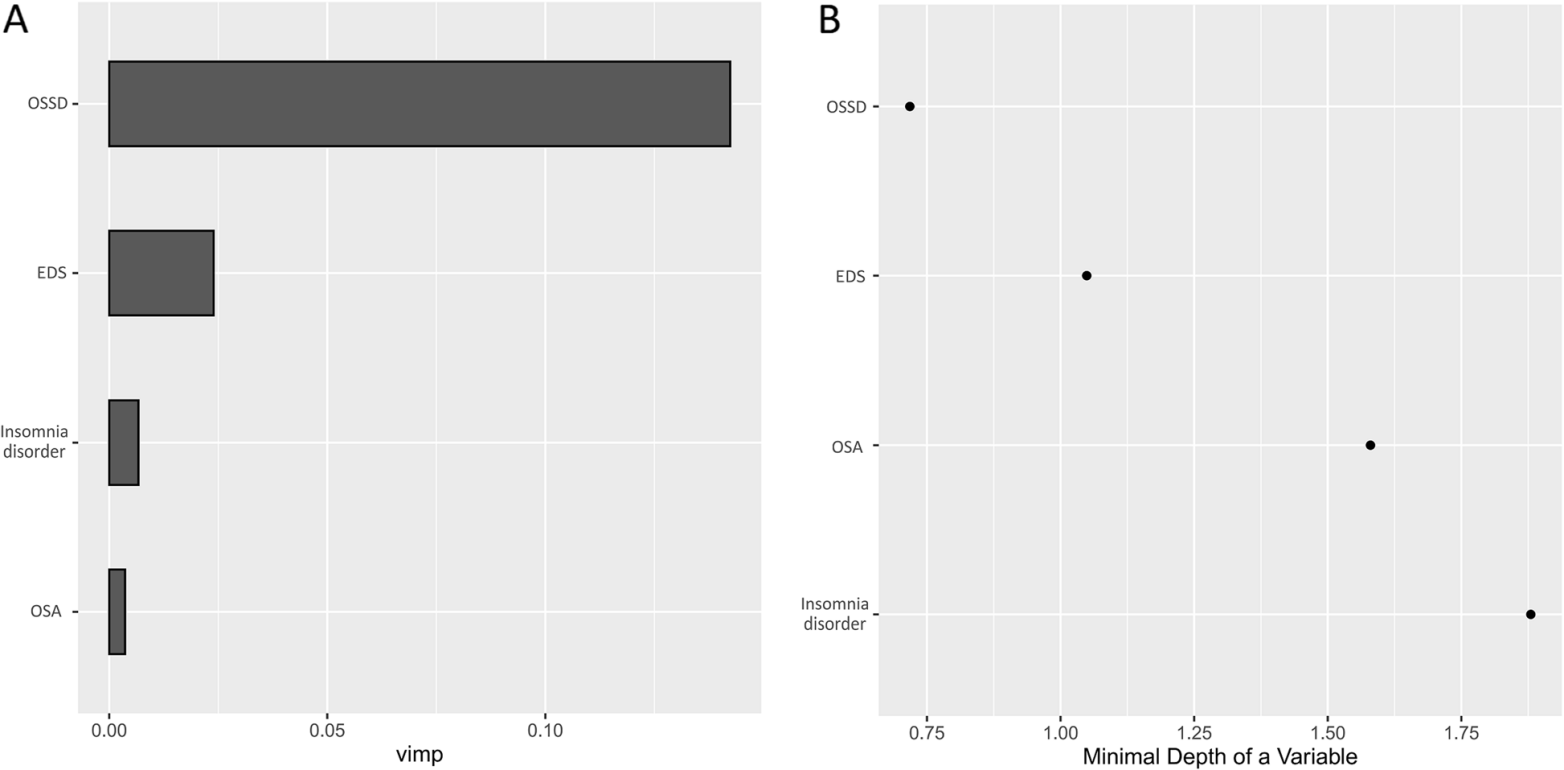
Relative importance of four sleep disorders for all-cause mortality in individuals with asthma by using machine learning with a random survival forest model. (A) Variable importance method; (B) minimal depth method

**Fig. 3:**
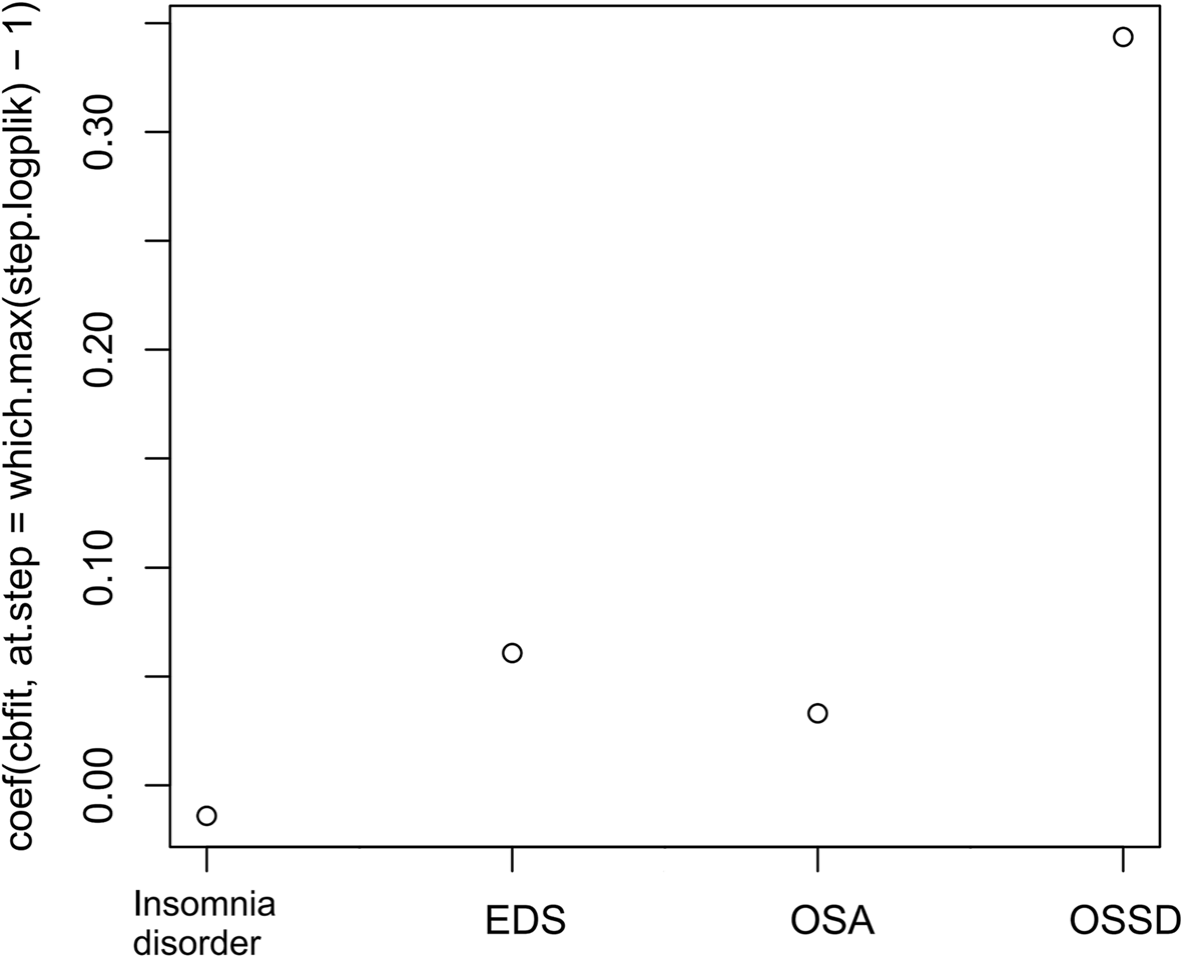
Standardized coefficient estimates of four sleep disorders for all-cause mortality in individuals with asthma by using machine learning with the CoxBoost model. The closer the standardized coefficient becomes zero, the less the effect of the variable on clinical outcomes

Random survival forest analyses in the training and validation datasets demonstrated that the relative importance of four sleep disorders for ACM followed a descending order of OSSD, EDS, probable insomnia, and OSA in the VIMP method (see Fig. 2A), while the rank from high to low was OSSD, EDS, OSA, and probable insomnia in the minimal depth method (see Fig. 2B). When considering all confounding factors, VIMP and minimal depth methods provided the integrated rank of relative importance as follows: OSSD, EDS, OSA, and probable insomnia. In CoxBoost analysis, the order of standardized coefficient of four sleep disorders for ACM was OSSD(0.344), EDS(0.061), OSA(0.033), and probable insomnia(0.014) in turn (see Fig. 3). When considering all confounding factors, the sequence of four sleep disorders made no change.

## Discussion

Our study simultaneously examined the associations between ACM and four sleep disorders in the asthma group and the no-asthma group. Our findings suggested that shortened objective sleep duration by means of polysomnography and severe EDS were associated with an increase in ACM in asthmatic patients. Similar results were not shown in OSA and probable insomnia. In general, machine learning demonstrated that OSSD of four sleep disorders had the largest contributor to ACM in asthmatics, followed by EDS. OSA and probable insomnia had no significant effect on ACM in individuals with asthma.

The first study from the Penn State Adult Cohort study reported that OSSD with subjective insomnia in men was associated with significantly higher mortality than that in men without short sleep duration and insomnia (adjusted OR = 4.0, 95% CI: 1.14, 13.99) [29]. When stratified by diabetes or hypertension at baseline, this difference was shown in the individuals with diabetes or hypertension but not in those without diabetes or hypertension. Fernandez-Mendoza et al. performed another four studies on the Penn State Adult Cohort study [30–33]. These studies suggest that the difference in ACM between OSSD and normal sleep duration was significant among the study population with hypertension [30], metabolic syndrome [31], cardiometabolic risk factors [32], and possible vascular cognitive impairment [33]. In the study of Bertisch et al. [34], a significantly higher risk of ACM was only shown in OSSD alone compared with normal sleep duration without insomnia (adjusted HR = 1.14, 95% CI: 1.01, 1.30, *P* = 0.04). This study indicated that the highest risk of ACM was shown in the shortest objective sleep duration group in three multivariate Cox regression and dose-dependent analyses. Interestingly, we failed to observe a similar relationship between objective sleep duration and ACM of the individuals without asthma in the same cohort based on statistical analysis (see Table 3). The impact of OSSD on ACM seemed to be much stronger in those with some underlying diseases at baseline, such as diabetes, hypertension and asthma. Compared with the population without asthma, asthmatics were associated with a higher risk of developing obesity and metabolic dysfunction (a cluster of at least three conditions that occur together, including obesity, increased hypertension, hyperglycemia and hyperinsulinemia, and dyslipidemia) [35]. Some studies using untargeted metabolomics approaches have further identified that asthmatics harbor several other metabolic dysfunctions, including oxidative stress and systemic inflammation [35]. Fernandez-Mendoza et al. [31] suggested that the effect of objective short sleep may be related to the degree of central autonomic and metabolic dysfunction. In the OSSD (< 6 h) group, more metabolic dysfunction was associated with a higher risk of ACM [31]. According to these findings, we speculate that (1) asthmatics are more likely to develop metabolic dysfunction during the long-term follow-up period than people without asthma and (2) OSSD among asthmatics with a high risk of metabolic dysfunction should be associated with a significant increase in ACM. The detailed and deep mechanism linking OSSD to an increased risk of ACM among asthmatic patients remains to be further studied.

A meta-analysis involving three studies showed that EDS had no significant relationship with ACM (pooled HR = 1.23, 95% CI: 0.94–1.61) when EDS was defined by ESS scores > 10 [36]. Our finding is that severe EDS but not all EDS is another independent risk factor for ACM in asthmatic patients. Unlike previous studies, EDS was divided into three levels according to ESS scores in our study. Similar to OSSD, individuals with severe EDS did not experience a higher risk of ACM than those without EDS in the no-asthma group (HR = 0.95, 95% CI: 0.71–1.28, *P* = 0.75, in model 3). Studies on EDS and asthma mainly focus on asthma control and the mutual influence of two diseases. The relationship between EDS and ACM in asthmatic patients needs more studies to be identified and explored. One study based on SHHS found that severe OSA was associated with higher ACM than the control group in the general population (adjusted HR = 1.46, 95% CI: 1.14–1.86). However, relatively fewer variables without EDS, OSSD and probable insomnia were limitations. The studies of Fernandez-Mendoza et al. [30–32] and Bertisch et al. [34] also included OSA and OSSD data but only considered OSA as an adjusted risk factor for mortality. Our study indicated that the presence and severity of OSA seemingly had no relationship with ACM among individuals with asthma after adjusting for more variables, which was similar to the result of Sumino et al. [19]. Studies on OSA and the risk of cause-specific mortality in asthmatic patients are still relatively few and are expected to increase. Two current meta-analyses found that different definitions of insomnia disorder are associated with inconsistent relationships with ACM [37, 38]. In this study, probable insomnia of four sleep disorders had the slightest influence on ACM in individuals with asthma, while it was an independent risk factor for ACM in people without asthma. The definition of probable insomnia was requested to simultaneously meet the symptoms and daytime consequences of insomnia; thus, the prevalence of probable insomnia was relatively lower than that in other asthmatic studies [8] and epidemiological surveys [10]. Document retrieval did not find a study about probable insomnia and mortality in asthmatic patients. The effects of OSA and probable insomnia on asthma control and development have been known for a long time; therefore, periodic screening and effective intervention of OSA and probable insomnia are crucial for the management of asthma.

The strength of our study is that we simultaneously examined the effects of four sleep disorders on ACM among asthmatics by using Cox regression and dose-dependent analyses. In addition, machine learning was first used to assess the importance of four sleep disorders for ACM in asthmatic patients. Nevertheless, our study also has some limitations. First, we need to realize the weaknesses of polysomnography, such as the “first night effect”. Second, we only estimated ACM among asthmatics due to the limitation of detailed causes of death. Whether OSSD results in asthma-related mortality remains to be further studied. Therefore, more large-sample prospective studies need to be conducted to assess the associations between cause-specific mortality among asthmatics and objective sleep duration and OSA. Third, as mentioned above, OSSD demonstrated 2.64-fold greater ACM than normal sleep duration among asthmatics. This effect was significantly stronger than that in the general population of Bertisch et al. [30]. We need to deeply explore the potential interaction between objective sleep duration and asthma. In addition, we did not obtain data about asthma control levels, which potentially affected our results.

## Conclusions

**O**ur findings suggest that a high risk of ACM among asthmatic patients is shown in OSSD and severe EDS but not in probable insomnia and OSA. Machine learning further confirmed the importance of OSSD and severe EDS for ACM in individuals with asthma. More large-sample studies are warranted to estimate the associations between cause-specific mortality among asthmatics and objective sleep duration and to deeply study the potential interaction between objective sleep duration and asthma.

## Data Availability

The data underlying this study were obtained from open SHHS databases. We obtained permissions to use the raw data from SHHS databases (https://sleep data.org/datasets/shhs/; accession number: hxq910813). The datasets generated during the study are available within the manuscript. The full datasets used in this analysis and the current study are not publicly available due to privacy concerns but are available from the corresponding author upon reasonable request.

## Abbreviations

AHI: Apnea hypopnea index
BMI: Body mass index
ESS: Epworth Sleepiness Scale
EDS: Excessive daytime sleepiness
FEV1: Forced expiratory volume in 1 second
FVC: Forced vital capacity
HR: Hazard ratio
obstructive sleep apnea: Obstructive sleep apnea
OSSD: Objective short sleep duration
OSA: Obstructive sleep apnea
SaO2: Oxygen saturation
SHHS: Sleep Heart Health Study.
